# Primary Care Academy: lessons learned from a large-scale innovative primary care project

**DOI:** 10.3389/fpubh.2024.1455810

**Published:** 2024-12-13

**Authors:** Manon Steurs, Emily Verté, Hans De Loof, Isabel Weemaes, Roy Remmen, Sibyl Anthierens, Patricia De Vriendt

**Affiliations:** ^1^Department of Family Medicine and Chronic Care, Vrije Universiteit Brussel, Brussels, Belgium; ^2^Laboratory of Physio Pharmacology, University of Antwerp, Antwerp, Belgium; ^3^Primary Care Academy, Antwerp, Belgium; ^4^Department of Family Medicine and Population Health, University of Antwerp, Antwerp, Belgium; ^5^Frailty in Ageing Research Group, Department Gerontology, Vrije Universiteit Brussel, Brussels, Belgium; ^6^Mental Health and Wellbeing Research Group, Vrije Universiteit Brussel, Brussels, Belgium; ^7^Research Center Care and Innovation, Artevelde University of Applied Sciences, Ghent, Belgium

**Keywords:** large-scale innovative primary care network, lessons learned, primary healthcare, achievements, development, implementation process

## Abstract

**Introduction and context:**

The social and healthcare system faces numerous challenges, with primary care playing a key role in achieving universal and equitable health coverage. However, the primary care field often struggles with limited research capacity, activity, and funding.

**The Primary Care Academy:**

To address these gaps, the Primary Care Academy (PCA) - a large-scale, innovative, interdisciplinary research and networking organization, encompassing then organization in the primary care field, was established and funded in 2019 by the Fund Dr. Daniel De Coninck, a charity foundation, managed by the King Baudouin Foundation, attempted to fil this gap.

**Objectives:**

The aim of this study was identifying lessons learned on the implementation and achievements of a large-scale, innovative research and network organization, specifically the PCA.

**Method:**

This study evaluates the PCA’s development process, focusing on its key achievements and critical elements by using a mixed-method data collection approach. After 5 years of rigorous collaboration, several lessons can be drawn regarding accomplishments and process flow, particularly in terms of a shared and clear vision, governance, leadership, and organizational culture.

**Discussion and conclusion:**

These lessons can inform future adaptations in the continuation of the PCA and serve as a guide for other caritative large-scale innovative initiatives.

## 1 Introduction and context

A multitude of concurrent challenges burden the current social and healthcare system (1), resulting in a growing number of people with long-term and complicated care needs (2, 3). This includes, for example, the aging population, a rise in chronic conditions, cognitive and functional impairments, mental health issues, and social vulnerability (2, 3). Combined with the rising health literacy and consciousness, and the preference to live and age healthily within one’s personal environment, the social and healthcare policies and practices are profoundly impacted. However, care delivery itself often lacks a comprehensive, person-centered approach (4–7). As primary care is designed to be the initial point of contact of the social and healthcare system, a pro-active, person-centered, community-based, and comprehensive primary social and healthcare approach may help to address these needs (7). Moreover, to create an inclusive care system that caters to the complex needs of individuals, a synergistic relation between informal and formal caregivers and care recipients, is essential (6). Consequently, government policies are shifting towards community-based integrated care practices (8) and primary care in Belgium, at both the federal and regional Flemish level, is adopting the Quintuple aim (9). The mandated reorganization of primary care resulted among other things in the establishment of 60 Primary Care Zones and the Flemish Institute for Primary Care (Vlaams Instituut voor de Eerste Lijn: VIVEL) in 2019 (10). The Primary Care zones aim to support the coordination and planning of care on a local level and help organize care services for larger groups of the population, whereas VIVEL aims to provide a continuous source of stimulus and expertise (10).

Primary care can play an essential role in the improvement of universal and equitable health coverage (11). While strong research is essential for driving effective reforms in primary care and ultimately delivering high-quality patient care (12), the field often struggles with limited research capacity, activity and funding (13, 14). The Fund Dr. Daniël De Coninck managed by the King Baudouin Foundation (KBF) acknowledges the shortage of funding of research in the interest of primary care in Belgium and therefore aims to support initiatives to improve to accessibility and quality of primary care. Hence, a call for an independent and coordinated consortium to carry out interdisciplinary research to strengthen primary care was issued. The call focused on three main elements: (i) increasing the visibility of research towards primary care, (ii) developing vision and carrying out research and innovation in primary care, and (iii) promoting networking between universities and universities of applied sciences, as well as between all relevant stakeholders. Thus, the Primary Care Academy (PCA) was established, a large-scale, innovative, and interdisciplinary research and networking organization, aiming to innovate primary care in Flanders and Brussels.

The development of integrated systems to handle complex challenges faced by decision-makers often necessitates interdisciplinary research including two or more disciplines (15–17). This is inherently complex to facilitate, manage, as well as evaluate (18). Hence, the question: what can we learn from 5 years of this innovative, large-scale, interdisciplinary network?

## 2 The Primary Care Academy

**Figure 1:**
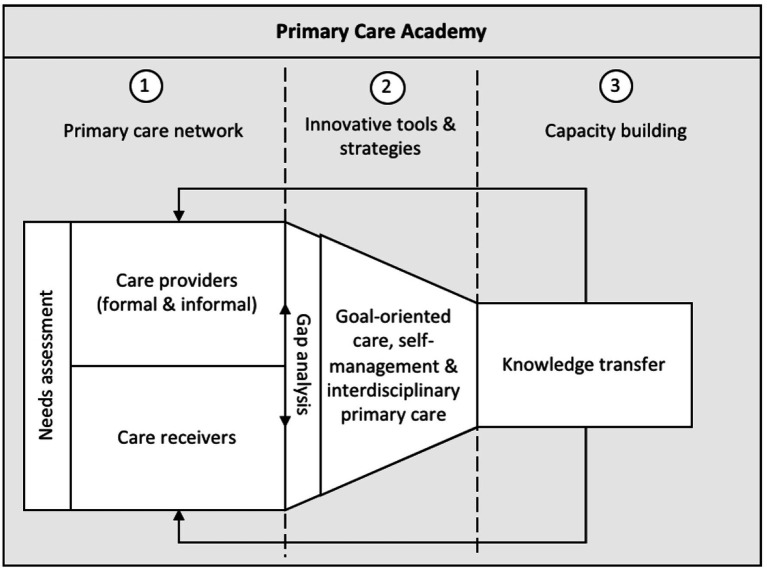
Objectives of the Primary Care Academy.

The PCA is a large-scale, innovative, and interdisciplinary research and networking organization, acting as a learning network. The collaborative serves as a hub for the collection, creation, dissemination, and implementation of knowledge. The main objective is to effectively cater to the care needs of individuals with moderately complex issues, within their informal and formal care settings in primary healthcare environments. This overarching goal was encapsulated by the formulation of three objectives of the PCA (Figure 1):

1. Build an interdisciplinary *primary care network* of teachers and researchers in primary care that reinforces state-of-the-art primary care practice in teaching and research.
2. Develop and implement *innovative tools and strategies* for proactive and person-centered primary care, built upon the principles of goal-oriented care, self-management and interdisciplinary primary care networks embedded in the community.
3. *Build capacity* of care receivers and formal and informal care providers in primary care, as well as researchers in primary care.

**Figure 2:**
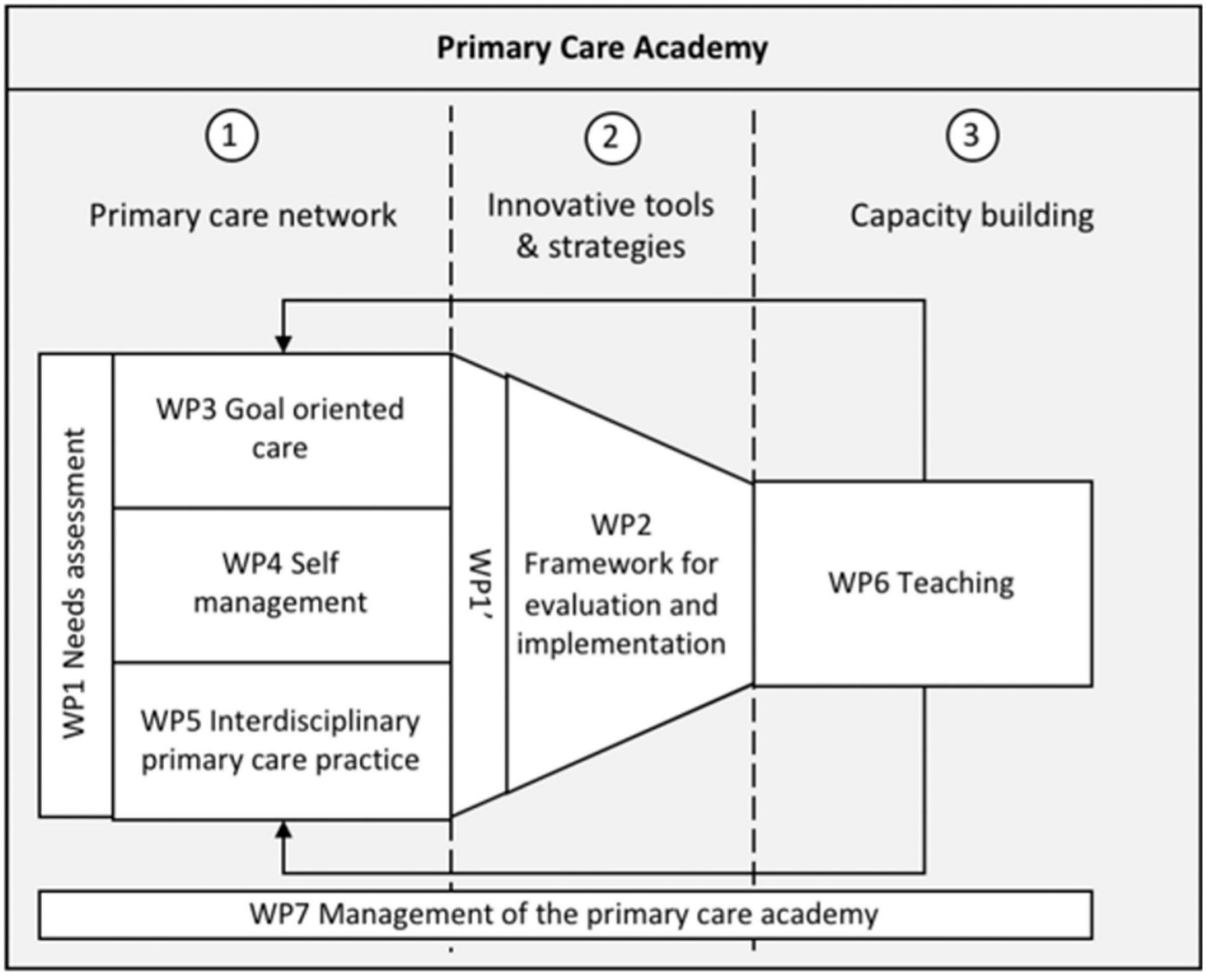
Research and teaching objectives of the PCA. WP stands for work package.

These overall goals were translated into a research plan. First, a review of the primary care context in Flanders, and a needs analysis, acting as the foundation for further research was conducted. Three focal topics were defined, acting as cornerstones to the PCA, namely (i) goal-oriented care, (ii) self-management and (iii) interprofessional collaboration (Figure 2). However, as the PCA was initiated in 2018 and established mid-2019, the beginning was vastly impacted by the Covid-19 pandemic, influencing its pre-established goals and operational dynamics.

The inclusion of various primary care stakeholders, both academic and non-academic, was a distinguishing characteristic of the PCA. Specifically, four universities and six universities of applied sciences, with 38 experts in more than 10 disciplines, along with societal partners such as the White-Yellow Cross and Flemish Patient Representatives were included. These stakeholders were structured into seven different research and teaching departments (delivering certain work packages or WPs), oriented towards one of the three objectives, based on an adapted version of the framework of the Medical Research Council (19). By the means of an intensive co-design process, an elaborate interdisciplinary project was developed, translating these objectives into operational targets with accompanying milestones. This facilitated a systematic user co-creation approach that integrated research and innovation. An auxiliary management department was established to guarantee seamless information flow and stakeholder engagement. By mediating, actively involving, and managing relations with stakeholder groups, the governance structure reinforced a multi-stakeholder approach. The PCA set out to implement participatory action research to foster a change of culture and engage stakeholders in collective learning, as well as a participatory process for creating and implementing decisions.

**Figure 3:**
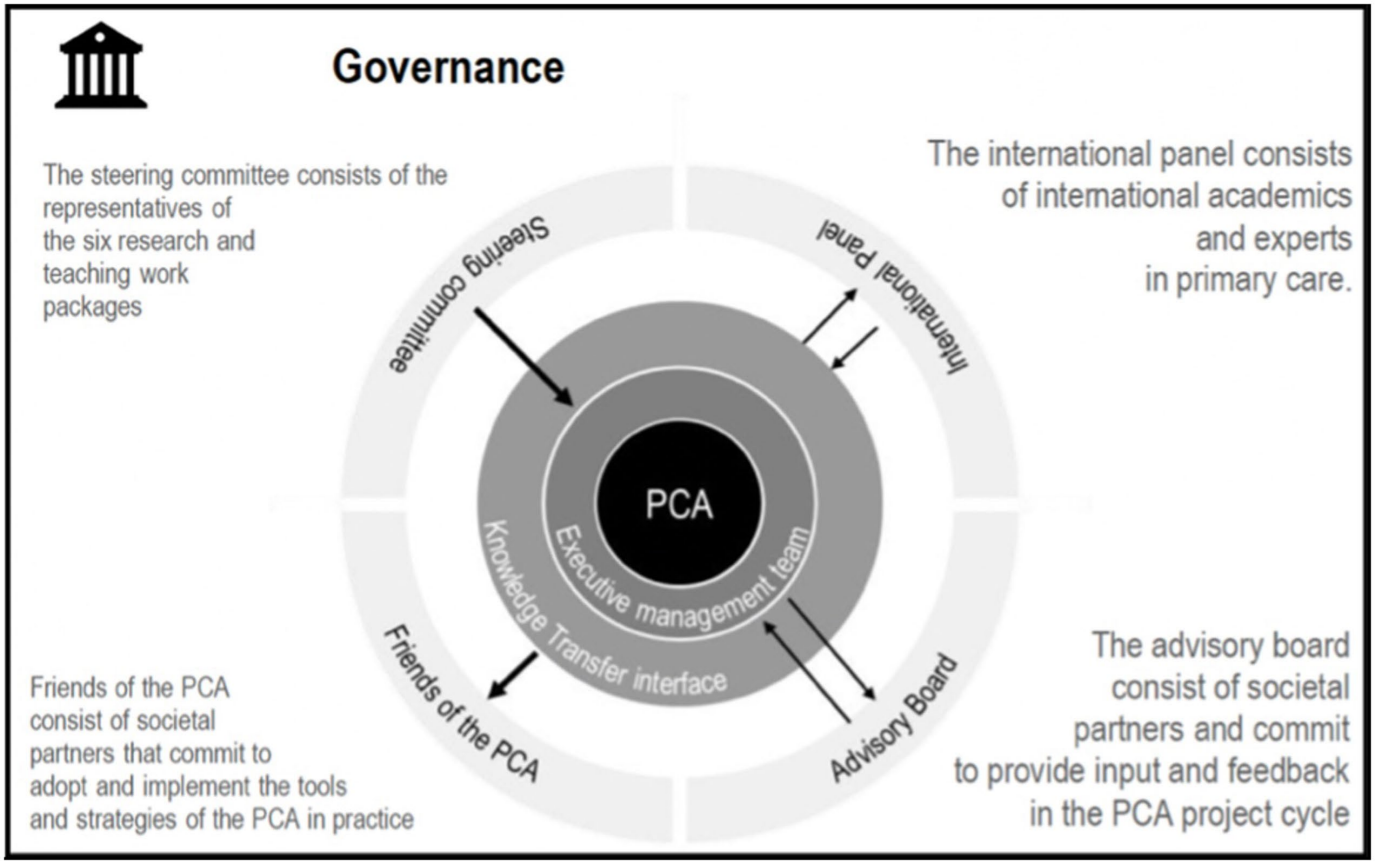
Governance framework of the PCA.

Stakeholders were included in the governance through the ‘Steering Committee’ and the ‘Advisory Board’, while the broader society was included through the ‘Friends of the PCA’ and the ‘International Panel’. Therefore, the PCA had extensive connections with networks of care recipients, care providers and professional organizations, resulting in a large variety of research data, infrastructures, and access to respondents. In the monthly Steering Committee, the process flow was monitored, as it exchanged information about the processes and outcomes of each department, made shared decisions, and safeguarded the general objectives. Initially, the PCA had an executive management team consisting of a senior and junior leader, responsible for the day-to-day tasks of running the partnerships, its strategic direction, and finances. The senior leader acted as a spokesperson (point of contact, executive of staff members, enforcer of household regulations, and person with final responsibility), while the junior leader was responsible for the daily coordination (right-hand person of the senior leader, focussing on operationalising). Figure 3 provides an overview of the governance framework of the PCA.

## 3 Method

This paper aims to formulate the lessons learned from the PCA’s development process, with a particular focus on identifying its key achievements and critical process elements. By applying a mixed method data collection method, various kinds of data were considered, allowing progressive insight in the achievements and the overall implementation process of a large-scale, innovative and interdisciplinary research and networking organization such as the PCA.

### 3.1 Data collection

A mixed-method data collection strategy was implemented, as both qualitative and quantitative data were included, stemming from multiple sources and evaluation moments.

#### 3.1.1 Qualitative data collection

During the qualitative data collection, several documents produced by the PCA were selected for a document analysis. Specifically, the project call and description and the five annual reports written by the PCA were included.

- *Project call and description*: documents containing all information regarding the objectives, the governance structure, and stakeholders of the PCA.
- *Annual reports*: yearly reports regarding progress made towards achieving goals and targets, drawn up by the executive management team, and approved by the KBF.

Additionally, the PCA performed internal follow-up focus groups to assess the experiences of its members regarding the process. Specifically, the following groups were questioned: (1) the Advisory Committee, (2) the Steering Committee, (3) the work packages, and (4) the Knowledge Transfer Interface. The results of the focus groups were described in reports, which were selected as qualitative data. Noteworthy, the PCA initiated these focus groups.

#### 3.1.2 Quantitative data collection

The quantitative data collection focused on information regarding publications, social activities and events, and social media presences of the PCA. Specifically, numbers of published papers and papers under review were gathered, as well as numbers of activities, followers on social media and subscribers to the newsletters.

### 3.2 Analysis

**Figure 4:**
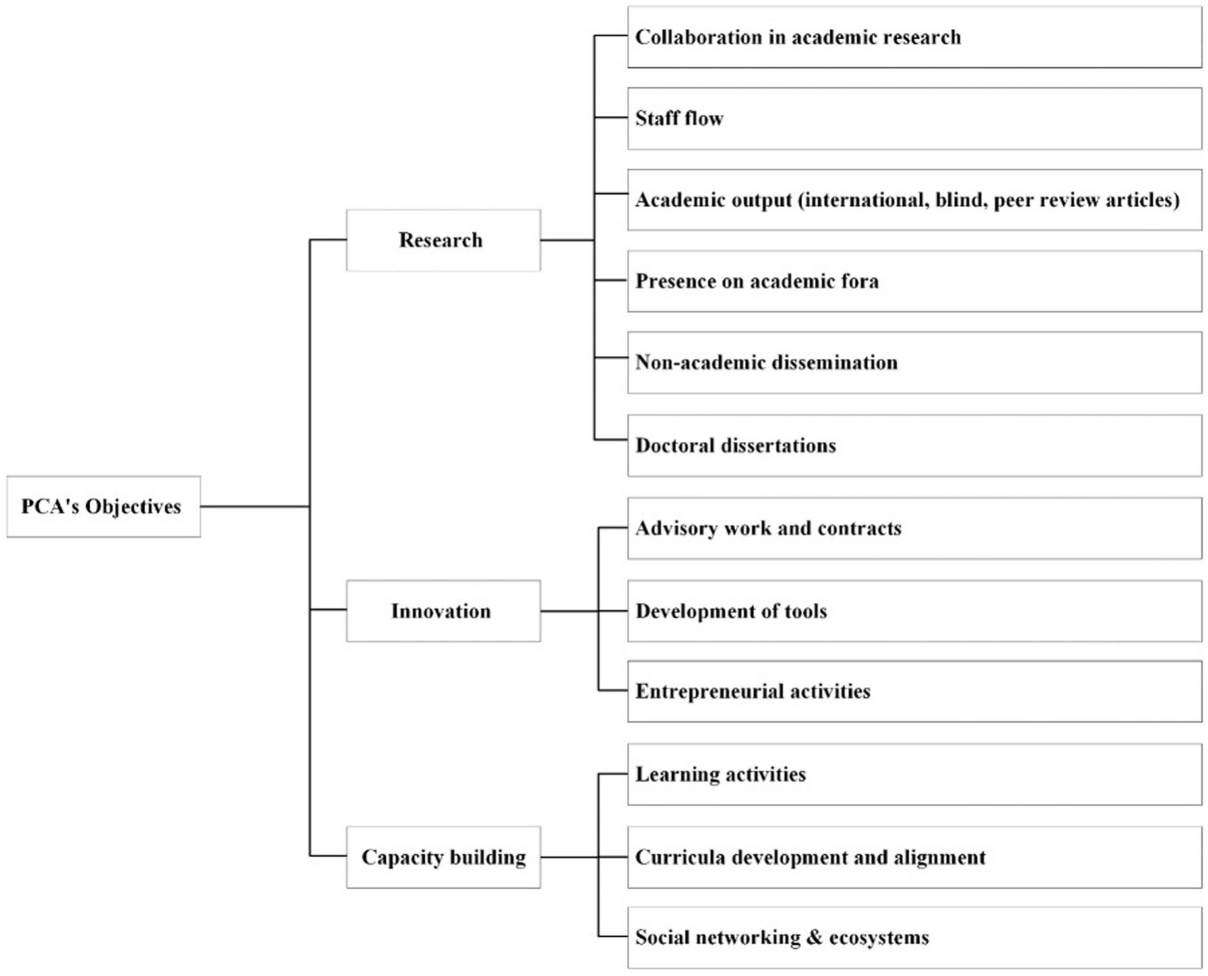
Main objectives and accompanying operational targets.

The data were analyzed, looking for achievements and process aspects of the PCA. To evaluate the achievements, several general targets for each objective were defined within the PCA after approximately two and a half years. The general targets were applicable to the context of the PCA, based on research from Molas-Gallart, Salter (20) and Bornmann (21) (Figure 4). These goals must be contextualized, with an understanding of the qualitative determinants that influence their achievement (21). To assess the process, the determinants of innovation in healthcare (22) were applied: (i) a clear and shared vision; (ii) structure, support and resourcing for innovation; (iii) leadership; (iv) organizational culture; and (v) organizational learning (22).

The evaluation was not systematically implemented, as the PCA lacked a clear action plan to execute the evaluation and improve on learnings at the start. Nonetheless, the obtained reports provide insight in the PCAs achievements and process over the past 5 years. The analysis of the data was executed by an external person (author 1) appointed with the task. The results of the analysis were presented to the co-authors (members of the PCA) who provided progressive insight in the achievements and the development and implementation process.

## 4 Discussion

Following 5 years of collaboration, numerous insights for the PCA can be discerned retrospectively, both in terms of how well pre-established goals (i.e., achievements) were met and the operational dynamics of the PCA (i.e., development and implementation process). These insights can be leveraged to steer the future direction of the PCA.

### 4.1 Achievements

As described above, the PCA presented three main objectives to accomplish (Figure 2), divided into operational targets and milestones (Figure 4), namely research, innovation, and capacity building.

#### 4.1.1 Research

Collaboration in academic research was established by the inclusion of minimum two academic partners of the PCA in each research and teaching department. Minimal staff turnover was reported, even though changing manpower (changing work, sickness, maternity leave) posed a challenge for the continuity. At the time of writing this article, the PCA reported on having a remarkable academic output with 14 published peer reviewed articles and others yet to come. Throughout the years, the PCA was present on different academic forums. For example, the four PhD candidates attended at least 16 conferences on behalf of the PCA. Regarding ‘non-academic dissemination’, the PCA reported to be on track, as the PCA organized one kick-off event, six ‘lunch & learn’ webinars, two summer courses, and two Belgian conferences in collaboration with the French-speaking chair. After feedback from an international board and the KBF, the focus of the PCA consequently shifted towards dissemination activities at an earlier stage, before the findings were published in international, peer-reviewed journals (13 published, and 6 under review), by using the ‘lunch & learn’ webinars and social media platforms, reaching more than 1,200 followers on LinkedIn and more than 1,000 subscribers via the newsletter. The PCA acknowledged facing challenges with their website in their annual reports. Nonetheless, these issues were actively addressed with the support of a newly appointed valorisation and communication manager together with the change and impact agent. Throughout the yearly follow-up reports, the PhD students were described as on track, with 3 doctoral dissertations successfully finalized before the end of the PCA, and 1 shortly after the end.

#### 4.1.2 Innovation

Throughout the 5 years of the PCA, steps were taken towards the development of tools for practice. However, as these need to be grounded in research, they are currently in various stages of implementation. The context in which research results are achieved and translated into practice, influence innovations (23). For example, the momentum the topic gained in academic research and practice: one PhD candidate researched self-management, which despite its interconnectedness with goal-oriented care, did not gain the same kind of momentum. Nonetheless, they managed to produce toolboxes regarding goal-oriented care, self-management, and interprofessional collaboration (24). Therefore, this aim was partially achieved, along with the entrepreneurial activities. Moreover, the PCA had the opportunity to collaborate with VIVEL, which has a mission to offer educational resources like tutorials and webinars to the public. The PCA developed a screening set to ensure the quality of the material. PCA researchers also developed and tested the interprofessional training for goal-oriented care for VIVEL. Additionally, since VIVEL aims to enhance the inclusion of the engagement of persons with a care and support need within the so-called ‘Zorgraden’, the PCA developed a tool kit to support this.

#### 4.1.3 Capacity building

The PCA worked towards the operational target of ‘social networking and ecosystems’. As indicated in the annual reports, the PCA has committed to the expansion of its network in the post-Covid-19 era. For example, the PCA managed to include 44 of the 60 Primary Care Zones in its network, and team members of the PCA and the PhD students tried to collaborate as much as possible in many key events with VIVEL, events by the KBF and other (inter)national events. Within the network of the PCA, partners seem to find each other and collaborate on writing proposals and articles or invite each other to give a guest lecture. On top of that, the PCA was active on LinkedIn, with more than 1,200 followers, and sent out biweekly newsletters, reaching 1,000 subscribers. To further enhance networking, the PCA organized two conferences. These events increased the sense of community among the participants. However, the development of ‘learning activities’ and ‘curricula development and alignment’ necessitate research outcomes which require time to develop. As a result, these objectives continue to pose a challenge.

### 4.2 The development and implementation process

In addition to evaluating the degree of attainment of operational objectives, the development and implementation process of the PCA offers valuable insights. However, healthcare innovation is determined by a complicated interaction of five different determinants, discussed below (22).

#### 4.2.1 A clear and shared vision

The diversity of partners and stakeholders involved has proven to be a challenge, as the PCA is a large network of people with differing backgrounds, disciplines, cultures, and languages. Bringing together a diverse range of perspectives, methods, and contexts, has been described as one of the biggest challenges of transdisciplinary research (25), as the participation of multiple stakeholders can complicate the process of defining the problem that the project aims to address (26). The strength of such a research or project stems from the gathering of a diverse group of perspectives, yet it also demands their convergence towards a shared objective (26). The development of a clear conceptual model can potentially help align research questions and objectives (27). Furthermore, effective adoption of care innovations can be hindered by a lack of a common language among primary care professionals from various backgrounds (14).

These difficulties characterized the first years of the PCA, as its internal and external diversity was substantial, and all involved parties had their own interests. Thus, during the first years, there has mainly been a focus on consolidating the research and teaching departments, demanding time and energy from everyone involved. The document analysis revealed that a bottom-up approach and an intense participatory decision-making process were intended to be applied, to create a shared vision and a common language. Although it proved to be a challenge, the PCA managed to take steps forward, improving brand awareness and a consolidation of market position on a macro (policy) level.

#### 4.2.2 Support, structure, and resourcing for innovation

Research indicates that implementing structures, processes, and routines can provide a stable framework that minimizes risk to management levels, thereby allowing interpersonal trust to grow and endure (28). Thus, the PCA implemented a versatile and intricate framework or governance model, fostering collaboration (Figure 3). For example, the executive management team originally consisted of a junior and a senior leader, which after feedback was expanded with complementary expertise, namely a communication manager in 2021 (internal and external communication, managing the website, social media, and the newsletter) and a valorisation and change maker agent in 2022 (developing tools and products based on research results). The collaboration between the different profiles was managed by introducing a weekly meeting to discuss the daily situation and adapt if necessary. Furthermore, the PCA was structured based on content, i.e., research topics were organized in five research departments and one teaching department (Figure 2), respectively focusing on knowledge creation and translation of science into practice. Although challenging, the PCA tried to support and facilitate cooperation between these departments through several activities, resulting in a variety of outcomes, such as events and workshops. One way in which the PCA and its members catered towards the challenges regarding knowledge valorisation, was by executing a needs assessment survey to establish the needs of the primary care network (29). This data provided insight into the context of primary care providers, which can influence the extent to which certain research topics and results are relevant and gain momentum. To ensure information flow between these departments, the PCA completed the full life cycle of innovation (30) by implementing the “Knowledge Transfer Interface” (Figure 3) and the dedicated department “Evaluation and implementation of primary care interventions.” Initially, a representative of each department was included in the Steering Committee and was supposed to guarantee information flow. However, concerns were raised regarding the representation of stakeholders not included in the Steering Committee. This original structure hampered information flow towards all the partners and undermined support for the PCA, influencing the dynamics within the departments and the Steering Committee.

Information flow requires adequate communication, which has been acknowledged as both a challenge (26), and an added value (22, 31–34). However, design elements, i.e., how the project is constructed to support cooperation and impact, are not equivalent to effective communication, shared knowledge, and a smooth flow of information (35). Consequently, after 1 year, the governance structure was modified to include a representative of all partners to remedy the challenge and respond to stakeholders’ concerns. The representation of all stakeholders in the Steering Committee ensured and improved information flow and support for the project. Furthermore, the executive management team met weekly. Additional activities were implemented to reshape internal communication channels, i.e., a document management system was implemented and the online ‘Weekly Wednesdays’ were introduced to report to the network regarding relevant primary care news. Although the members of the PCA described the implementation of these tools as beneficial to internal and external communication, they were never officially evaluated. The members expressed their need for (more) informal and formal communication to enhance alignment between the different departments. The PCA, therefore, organized meetings and network moments where people were able to get to know each other, enabling further collaboration between the members. However, the Covid-19 pandemic resulted in a lack of opportunities to meet physically as a team. After mere 6 months of building connections and starting up collaborations, both internal and with external partners, this pandemic forced the PCA to move all their meetings, social and research activities online. Nonetheless, it accelerated working digitally and convening meetings with everyone involved became easier. Additionally, this challenge prompted two of the PhD candidates to write a paper on digital focus groups (36).

The Covid-19 pandemic characterized the beginning of the PCA, which resulted in high stress levels, influencing researchers and collaboration (37, 38), along with the day-to-day primary care practices (39). For example, developing new collaborations with the ELZ and VIVEL was challenging, as the Covid-19 pandemic emerged before they could get properly organized, forcing primary care professionals and providers to focus on testing and vaccinations. Most problems arose in the research departments, in which input from the field was needed (29). Such environmental factors, in conjunction with the academic and policy settings, impeded cooperation across disciplinary and institutional boundaries. Although the influence of Covid-19 on the first term of the PCA was vast, the PCA and its members were able to adapt to this new context and progress towards their predefined research plan.

The PCA recognised this challenge and addressed this by investing an equal amount of funding in the four functions of the network: (i) gathering, (ii) creating, (iii) translating and guiding, and (iv) valorising knowledge. This division was helpful in dividing tasks and responsibilities, as well as in remedying concerns regarding collaboration across boundaries. Although the PCA made this division, the four functions were interrelated, as the research department provided knowledge to translate into practice for the teaching department. However, carrying out research takes time (40), thus influencing and limiting the work and results of the teaching department within the 5 years.

#### 4.2.3 Leadership

A couple of leadership tasks can be distinguished in the literature: (i) structural leadership tasks, (ii) process tasks, and (iii) cognitive tasks (41). Structural leadership tasks deal with the managing issues of coordination and information exchange (41), and were discussed above.

The process tasks deal with ensuring constructive and productive interactions among team members (41). Due to the diversity characterizing the PCA, managing the involved stakeholders became an essential task of the leadership. Navigating diversity and addressing complex, intertwined agendas appears to be necessary to work towards a shared goal (26), and represents a significant challenge. The PCA implemented stakeholder management, to align the goals of the various stakeholders involved in the internal network, along with the societal partners. The development of a clear conceptual model can potentially help align research questions and objectives (27), and laying out different responsibilities between involved researchers can effectuate ownership (18). Since trust can be understood as a prediction of reliance, derived from what each party knows about the others (42) having a clear set of goals, roles, and responsibilities, proved to be beneficial. For example, the universities took the lead in the research departments, while the universities of applied sciences took the lead in the teaching department. Although the cooperation between universities remained a challenge, a clear set of goals, roles, and responsibilities within the research group enabled them to progress towards achieving targets. In comparison, the goals for the teaching department were less well defined in the project description, therefore focussing and developing them took obviously longer. Due to this shortage, their progress towards achievements was hampered. The document analysis revealed feelings of mistrust when leadership tried to intervene, support, or monitor the process with the general trajectory of the PCA in mind. Therefore, research suggests finding facilitators with the appropriate skillset to chair and guide the social process in a meeting where people have opposing ideas and clashes of culture (43). Furthermore, the face-to-face and online meetings and network moments were established to enhance communication between its members and address the underlying trust issues, since research accentuates the significant contribution of sharing critical information and having a high level of communication through continuous interaction to developing trust in high-performing teams and within business ecosystems (44). Moreover, communication processes are key mechanisms for cultivating trust (45), and are influencing every step of knowledge creation and learning maximalisation (46).

Tasks dealing with meaning-making through a mental mindset or model, are defined as cognitive tasks (41). Some members reported not feeling like an equal partner, referring to power dynamics at play between the various stakeholders. Although the PCA intended to keep building trust between all involved, considerable competition between academic institutions was reported. Scholars indicate that power asymmetries, as perceived by the involved parties, can vary due to the individual power differences (caused by, e.g., gender, age, educational background) in various cultural contexts and act as a barrier to collaboration (35). Research recognises the challenges that arise from the diversity of stakeholders involved in an interdisciplinary project but is not able to provide the most suitable methodology to address the tension, except by being aware of its presence throughout the various stages of the project (26).

Developing trust constitutes a challenge (26), but appears to be a crucial element to collaboration (33, 47), as inter- or transdisciplinary research teams depend on both the skills of the team members, and the trust and mutual respect among them (48). Although the PCA recognised the trust issues experienced by its members and implemented a few structural changes to address both power asymmetries and trust issues, they acknowledge their lack of time and attention spent towards these issues. No specific trust-building exercises or conflict-resolution protocols were introduced. The leader is responsible for intentionally planning a culture of trust, in which everyone is bound by a shared destiny (49). Moreover, leaders can, through their vision and goal setting, enable innovation by promoting organization values that encourage innovation, providing resources, and creating a climate which encourages innovation (22). To adjust adequately when confronted with challenges, scholars suggest the project description should allow time and space for reflection and social learning (50), as it provides the opportunity to learn from mistakes (26). Hegger, Lamers (50) describe a project leader who is a grounded and reflexive practitioner as the appropriate leader of a transdisciplinary research project (50), indicating experience in transdisciplinary research “can facilitate the process and manage tensions” (26). Therefore, there is a need for clear role descriptions with the necessary competencies to adequately manage a large-scale, multi-stakeholder project. In addition, flexibility is mentioned as a leadership skill in the context of project management (22). However, recent studies suggest clinical leadership, identifying all members of a healthcare team as potential leaders (51), as the concept is not exclusive to any domain or particular professional group (52).

#### 4.2.4 Organizational culture

Design features (i.e., how a project is constructed to support cooperation and impact), can be undermined by its relational (i.e., interpersonal and interinstitutional dynamics) and systematic features (i.e., pre-existing biases and norms which influence how the design and relational features take shape) (35). As the PCA was a unique and innovative organization, established in 2018 and initiated in mid-2019, there was not yet a strong bond, heritage, or tradition connecting its members, hampering extensive collaboration. Additionally, the institutional cultural differences caused tension. For example, the PCA supported and funded research of four PhD students, during which the universities and universities of applied sciences needed to work together as supervisors of their trajectory towards a doctoral dissertation. While this process was successful for the PhD students, stimulating collaboration and mutual learning, the institutional culture differences became apparent. For example, the universities focused on research with the intent to publish in international, peer review journals, while the universities of applied sciences focused more on teaching and doing research to publish in non-international, non-scientific journals.

Although the PCA was established with the intent to innovate, carrying out research does not automatically lead towards societal changes. Hence, the full life cycle of innovation (30) was implemented, encompassing collaboration across boundaries, outside of the comfort zone of the people involved. However, it is not common in academia to focus on immediate real-life impact and the production of tools and products. Thus, there was a need for someone with the capacity to translate and validate findings into tools and products. Therefore, a professional with the capacity for the valorisation of knowledge was contracted, i.e., someone with knowledge of the development of tools or products (valorisation and change agent). This resulted in the development of, among others, webinars and training modules, such as an education module on self-management.

#### 4.2.5 Organizational learning

The PCA can be perceived as a large-scale cross-cultural collaboration project, stimulating the cross-fertilization of its members. Within its network, thirty-eighth experts from various disciplines collaborate. For example, a minimum of two academic partners collaborated in each research department. This diversity was amplified by the backgrounds, the variety of years of service (senior/junior), geographical distribution (covering Flanders and Brussels), and gender. This way, the PCA hoped to create an open innovation culture, encouraging personal development and well-being, and therefore improve the overall team performance. Additionally, the implementation of annual network meetings boosted the learning culture the PCA wanted to provide.

## 5 Conclusion

The PCA was established as a large-scale innovative project with the aim to innovate primary care in Flanders and Brussels to effectively address the care needs of people with moderately complex problems and their informal and formal care context. This was divided into objectives regarding research, innovation, and capacity building. Through the design of the PCA, there was a clear focus on the involvement of stakeholders of primary care in Flanders and Brussels, applying a participatory decision-making process. Noteworthy, for the past and next 5 years, the PCA was and remains the result of philanthropy, which is not inherently sustainable. After a period of 5 years, we have the opportunity to reflect on our accomplishments and the workflow, which will enable us to learn and modify the PCA for future years. Overall, the operational targets of the three objectives were met, indicating that the PCA was able to adequately address challenges. However, a couple of lessons learned regarding the process were identified which can guide the second term of the PCA and improve its sustainability.

First, the distinction between the research and teaching departments posed a challenge. Therefore, the consolidation of, and the integration of the research and teaching departments into ‘academic workplaces’ ensures a basis for the next phase of the PCA, as it will solidify the clear and shared vision of the PCA. In these academic workplaces, the collaboration between research and teaching actors, along with stakeholders and citizens will be facilitated. By incorporating all actors in academic workplaces, the PCA expects to counter power asymmetries. To further enhance cooperation within these academic workplaces, the PCA will be implementing trust-building exercises during the first meetings. On one hand, the second term of the PCA can continue to build on this position and further expand its network of societal partners, as more stakeholders and citizens are introduced in the societal board. On the other hand, the PCA can enhance brand awareness on multiple policy levels. In the analysis of governance, the criticality of a flexible governance framework is vital for fostering, preserving, and adjusting the support and dialogue among a heterogeneous group of stakeholders.

A comprehensive set of tasks and roles should be established at the beginning of a trajectory, for everyone involved, along with a clear work plan to enhance collaboration. Leadership tasks and responsibilities should be solidified in an enforceable mandate, divided into multiple people with the appropriate skills, e.g., (i) someone who presents themselves as the face of the PCA, (ii) someone with a business profile (i.e., someone with the capacity for the valorisation of knowledge), (iii) a day-to-day manager (coordinator), (iv) a stakeholder manager, and (v) a communication manager. This way, all different, extensive tasks connected to the PCA can be assigned to the persons with the appropriate knowledge and capabilities to execute their roles. Therefore, the PCA will introduce household regulations, consisting of an overview of all involved actors with their accompanying tasks and responsibilities (i.e., job descriptions), and protocols to deal with challenges such as conflict and trust issues.

The PCA provided an environment in which people could get to know each other and other disciplines, functioning as a learning network. Although challenged by the Covid-19 pandemic, the PCA was able to stimulate connection by organizing network moments and encouraging cooperation. In addition, these activities can counter challenges between stakeholders, such as tension and feelings of distrust, when underlying agendas are acknowledged. Nonetheless, it remains essential to further engage all involved actors to take part in the learning collaborative of the PCA. Therefore, an additional structure to support collaborative learning will be introduced, appointing one person as the dedicated coach.

The PCA acknowledged the absence of quantitative data on the tools and activities used during the first term. Consequently, assessing the sustainability of both past and future activities has been identified as a key challenge for the PCA in the coming years. Implementing an evaluation framework, including tools and measurement systems, can enhance collaboration and information flow. Therefore, at the start of the PCA’s second term, an evaluation framework was introduced to systematically gather information and experiences, providing progressive insights into achievements and the development process. Adjusting the PCA according to the lessons learned from the first term and monitoring the next 5 years, provides a basis to improve the PCA and its sustainability. Additionally, the PCA will be developing a phased plan to enhance its sustainability in the future during the next 5 years. Specifically, it will be engaging with governmental bodies such as the Department of Care and the Policy Research Center, and with the Primary Care Zones and VIVEL, to increase the sustainability of the organization and its activities.

### Limitations

The results of this research stem from follow-up and annual reports, as well as group and individual interviews. Although many people participated, the evaluation of the PCA was not systematically established. Specifically, no external evaluator was appointed to conduct the interviews. The analysis was performed by an outsider of the Academy and the diverse team of authors commented on the results, later validated by the entire team of the PCA.

## Data Availability

The raw data supporting the conclusions of this article will be made available by the authors, without undue reservation.

## Group member of The Primary Care Academy

R. Remmen: Department of Primary Care and Interdisciplinary Care, Faculty of Medicine and Health Sciences, University of Antwerp, Antwerp, Belgium. E. Verté: Department of Primary Care and Interdisciplinary Care, Faculty of Medicine and Health Sciences, University of Antwerp, Antwerp, Belgium; Department of Family Medicine and Chronic Care, Faculty of Medicine and Pharmacy, Vrije Universiteit Brussel, Brussel, Belgium. M. M. Sirimsi: Centre for Research and Innovation in Care, Faculty of Medicine and Health Sciences, University of Antwerp, Antwerp, Belgium. P. V. Bogaert: Workforce Management and Outcomes Research in Care, Faculty of Medicine and Health Sciences, University of Antwerp, Belgium. H. De Loof: Laboratory of Physiopharmacology, Faculty of Pharmaceutical Biomedical and Veterinary Sciences, University of Antwerp, Belgium. K. V. den Broeck: Department of Primary Care and Interdisciplinary Care, Faculty of Medicine and Health Sciences, University of Antwerp, Antwerp, Belgium. S. Anthierens: Department of Primary Care and Interdisciplinary Care, Faculty of Medicine and Health Sciences, University of Antwerp, Antwerp, Belgium. I. Huybrechts: Department of Primary Care and Interdisciplinary Care, Faculty of Medicine and Health Sciences, University of Antwerp, Antwerp, Belgium. P. Raeymaeckers: Department of Sociology, Faculty of Social Sciences, Faculty of Social Sciences, University of Antwerp, Belgium. V. Buffel: Department of Sociology, Centre for Population, Family and Health, Faculty of Social Sciences, University of Antwerp, Belgium. D. Devroey: Department of Family Medicine and Chronic Care, Faculty of Medicine and Pharmacy, Vrije Universiteit Brussel, Brussel. B. Aertgeerts: Academic Centre for General Practice, Faculty of Medicine, KU Leuven, Leuven; Department of Public Health and Primary Care, Faculty of Medicine, KU Leuven, Leuven. B. Schoenmakers: Department of Public Health and Primary Care, Faculty of Medicine, KU Leuven, Leuven, Belgium. L. Timmermans: Department of Public Health and Primary Care, Faculty of Medicine, KU Leuven, Leuven, Belgium. V. Foulon: Department of Pharmaceutical and Pharmacological Sciences, Faculty Pharmaceutical Sciences, KU Leuven, Leuven, Belgium. A. Declerq: LUCAS-Centre for Care Research and Consultancy, Faculty of Social Sciences, KU Leuven, Leuven, Belgium. N. Verhaeghe: Research Group Social and Economic Policy and Social Inclusion, Research Institute for Work and Society, KU Leuven, Belgium. D. V. de Velde: Department of Rehabilitation Sciences, Occupational Therapy, Faculty of Medicine and Health Sciences, University of Ghent, Belgium; Department of Occupational Therapy, Artevelde University of Applied Sciences, Ghent, Belgium. P. Boeckxstaens: Department of Public Health and Primary Care, Faculty of Medicine and Health sciences, University of Ghent, Belgium. A. De Sutter: Department of Public Health and Primary Care, Faculty of Medicine and Health sciences, University of Ghent, Belgium. P. De Vriendt: Department of Rehabilitation Sciences, Occupational Therapy, Faculty of Medicine and Health Sciences, University of Ghent, Belgium; Frailty in Ageing (FRIA) Research Group, Department of Gerontology and Mental Health and Wellbeing (MENT) research group, Faculty of Medicine and Pharmacy, Vrije Universiteit, Brussels, Belgium; Department of Occupational Therapy, Artevelde University of Applied Sciences, Ghent, Belgium. L. Lahousse: Department of Bioanalysis, Faculty of Pharmaceutical Sciences, Ghent University, Ghent, Belgium. P. Pype: Department of Public Health and Primary Care, Faculty of Medicine and Health sciences, University of Ghent, Belgium; End-of-Life Care Research Group, Faculty of Medicine and Health Sciences, Vrije Universiteit Brussel and Ghent University, Ghent, Belgium. D. Boeykens: Department of Rehabilitation Sciences, Occupational Therapy, Faculty of Medicine and Health Sciences, University of Ghent, Belgium; Department of Public Health and Primary Care, Faculty of Medicine and Health Sciences, University of Ghent, Belgium. A. V. Hecke: Department of Public Health and Primary Care, Faculty of Medicine and Health Sciences, University of Ghent, Belgium; University Centre of Nursing and Midwifery, Faculty of Medicine and Health Sciences, University of Ghent, Belgium. P. Decat: Department of Public Health and Primary Care, Faculty of Medicine and Health Sciences, University of Ghent, Belgium. R. Roose: Department of Social Work and Social Pedagogy, Faculty of Psychology and Educational Sciences, University Ghent, Belgium. S. Martin: Expertise Centre Health Innovation, University College Leuven-Limburg, Leuven, Belgium. E. Rutten: Expertise Centre Health Innovation, University College Leuven-Limburg, Leuven, Belgium. S. Pless: Expertise Centre Health Innovation, University College Leuven-Limburg, Leuven, Belgium. V. Gauwe: Department of Occupational Therapy, Artevelde University of Applied Sciences, Ghent, Belgium. D. Reynaert: E-QUAL, University College of Applied Sciences Ghent, Ghent, Belgium. L. V. Landschoot: Department of Nursing, University of Applied Sciences Ghent, Ghent, Belgium. M. L. Hartmann: Department of Welfare and Health, Karel de Grote University of Applied Sciences and Arts, Antwerp, Belgium. T. Claeys: LiveLab, VIVES University of Applied Sciences, Kortrijk, Belgium. H. Vandenhoudt: LiCalab, Thomas University of Applied Sciences, Turnhout, Belgium. K. De Vliegher: Department of Nursing – Homecare, White-Yellow Cross, Brussels, Belgium. S. Op de Beeck: Flemish Patient Platform, Heverlee, Belgium.
